# Influence of Ingestion of Eicosapentaenoic Acid-Rich Fish Oil on Oxidative Stress at the Menstrual Phase: A Randomized, Double-Blind, Placebo-Controlled, Parallel-Group Trial

**DOI:** 10.1089/whr.2022.0003

**Published:** 2022-07-13

**Authors:** Yoshihiko Mano, Ayaka Kato, Nobuo Fukuda, Keiko Yamada, Kenichi Yanagimoto

**Affiliations:** ^1^ Department of Sports Nutrition, School of Physical Education, Sendai University, Sendai, Japan.; ^2^ Food Function R & D Center Nippon Suisan Kaisha, Ltd., Tokyo, Japan.; ^3^ Department of Health and Welfare, School of Physical Education, Sendai University, Sendai, Japan.; ^4^ Department of Physical Education, School of Physical Education, Sendai University, Sendai, Japan.

**Keywords:** docosahexaenoic acid, eicosapentaenoic acid, menstrual cycle, oxidative stress, reactive oxygen metabolites (d-ROMs)

## Abstract

**Background:**

This study examines the effect of the supplements on the redox reaction in menstrual cycle. Participants took eicosapentaenoic acid (EPA)-rich fish oil supplements over two menstrual cycles.

**Materials and Methods:**

For this randomized, double-blind, placebo-controlled trial, 21 female members of a university basketball team were selected. Participants were allocated into the EPA/docosahexaenoic acid (DHA) group (EG, *n* = 11) and control group (CG, *n* = 10) through stratified randomization. The EG and CG took 3600 mg fish oil (containing 900 mg EPA and 403 mg DHA) and 3600 mg corn oil (without EPA and DHA), respectively, every day for two menstrual cycles. The redox reaction was measured four times: the menstrual and follicular phases in two menstrual cycles.

**Results:**

There was a significant difference in reactive oxygen metabolites (d-ROMs) and potential antioxidant capacity during the menstrual phase by the main effect of time (before and after intake) in EG and CG (*p* < 0.01). In a subsequent test, d-ROMs were significantly lower after intake in EG and CG (*p* < 0.05); however, no significant difference in potential antioxidant capacity was found. A significant difference was noted in d-ROMs and potential antioxidant capacity during the follicular phase by the effect of time (before and after intake) only in EG (*p* < 0.01). Significant decreases in d-ROMs and increases in potential antioxidant capacities were observed after intake (*p* < 0.05).

**Conclusion:**

EPA-rich fish oil supplementation over two menstrual cycles demonstrated active involvement in the antioxidant function during menstrual and follicular phases.

The protocol was registered at the University Hospital Medical Information Network Clinical Trial Registry (registration no. UMIN000028795).

## Introduction

Menstrual cycle affects female athletes' performance as well as their cardiopulmonary functions, such as exercise tolerance and energy substrate.^1^ In addition to changes in the metabolic function caused by the menstrual cycle, increased protection against oxidative stress reaction from inflammatory stress during exercise or the menstrual cycle is necessary. During exercise, the oxygen consumption by the body increased by >10 times than that during rest. The generation of active oxygen and free radicals increases to a similar extent during exercise. The effect of oxidative stress on athletes is as follows: exhaustive exercise increases the production of reactive oxygen species (ROS). In the absence of antioxidants, oxidative injuries will occur, such as muscle weakness and fatigue.^2^

Regarding the relationship between menstrual cycle and oxidative stress, menstrual cycle is divided into the menstrual, follicular, and luteal phases. The secretion of estrogen, a female hormone, is highest during the follicular phase, and estrogen has an antioxidant effect that eliminates active oxygen.^1,3^ Conversely, nitric oxide, a free radical, is produced with the secretion of estrogen. The menstrual cycle might affect the redox balance. Therefore, we assumed that in female athletes, excessive oxidative stress or redox imbalance are caused by hormonal secretion due to the menstrual cycle and exercise-induced inflammation. Changes in oxidative stress owing to the menstrual cycle as well as the effect of the menstrual cycle on the redox reaction during exercise have been described in previous studies on the general population.^4–7^

Furthermore, female athletes have significantly higher markers of oxidative stress than the general female population.^8^ Of the previous studies, we focused on the report of Cornelli et al^4^ and observed changes in oxidative stress due to menstrual cycles in female athletes. Thus, we focused on eicosapentaenoic acid (EPA), a fish oil component, as it was found to be associated with the suppression of oxidative stress, inflammation, postexercise test inflammation, and antioxidant potential in athletes,^9–11^ and examined the effects of EPA-rich fish oil supplements on the suppression of oxidative stress. This study examined the redox reaction during menstrual cycle and the effect of EPA intake on the redox reaction for each menstrual cycle in female athletes.

In addition, because EPA intake eases dysmenorrhea,^12–14^ we examined the subjective scale of dysmenorrhea and premenstrual syndrome (PMS) before and after the intake of the trial food. We hypothesized that the intake of supplements with high EPA content suppresses oxidative stress during menstrual and follicular phases.

## Materials and Methods

### Ethics statement

This study was conducted in accordance with the guidelines of the Declaration of Helsinki. The protocol was registered at the University Hospital Medical Information Network Clinical Trial Registry. This study was approved by the Ethical Review Board of the University of Sendai (approval no. 29-06). All participants provided verbal informed consent. Participants were informed in advance of the purpose, methods, and privacy protection of the study, and written consent for participation was obtained. Joint researchers of the same gender as participants recorded and confirmed information related to menstrual cycle.

### Participants

The participants were 23 female university students who were part of the basketball team. Twenty-one students with confirmed regular menstrual cycles were selected. A regular menstrual cycle was defined as having the following three items: two cycles between 25 and 38 days, a basal temperature confirmed based on the low- and high-temperature phases, and a positive result for luteinizing hormone. For the basal body temperature, information about menstrual cycle was recorded by the group ahead of time; the basal body temperature from two menstrual cycles was used to confirm the low- and high-temperature phases. Terumo Electronic Thermometer WOMAN C502 (Terumo Corporation, Japan) was used to measure basal body temperature.

**FIG. 1.**
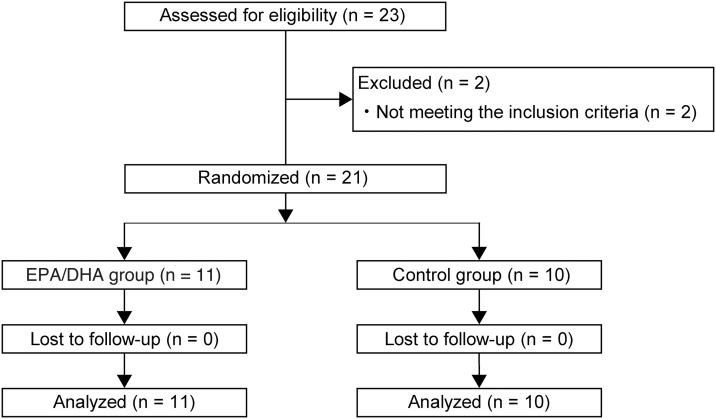
Flow diagram of progress at each stage of a randomized, double-blind, placebo-controlled trial (i.e., enrolment, intervention allocation, follow-up, and data analysis).

For the positive determination of luteinizing hormone, the ovulation day was predicted from menstrual cycle, and a positive determination for the two menstrual cycles was made using a self-test kit for luteinizing hormone (Mizuho Medy Co., Ltd., Japan); a female joint researcher made this determination. Those with gynecological diseases or severe menstrual symptoms requiring sedatives; those taking other drugs or supplements or smokers; and those with allergies to bluefish, such as sardines and mackerel, were excluded from the study. A flowchart showing the process of each step in this trial is shown in Figure 1.

### Supplementation

Supplementation was prepared considering the safety based on previous studies.^9,15–18^ A 300-mg soft capsule was made with the following fat components: fish oil with high EPA content or corn oil as a placebo (Nippon Suisan Kaisha, Ltd., Tokyo, Japan). The participants took 12 supplemental capsules per day, dividing them equally after each meal. The EPA/DHA group (EG) took 3600 mg of fish oil (900 mg of EPA and 403 mg of DHA) per day, and the control group (CG) took 3600 mg of corn oil (without EPA and DHA) per day. There were no specific instructions provided by us regarding the diet during the experiment.

### Experimental design

**FIG. 2.**
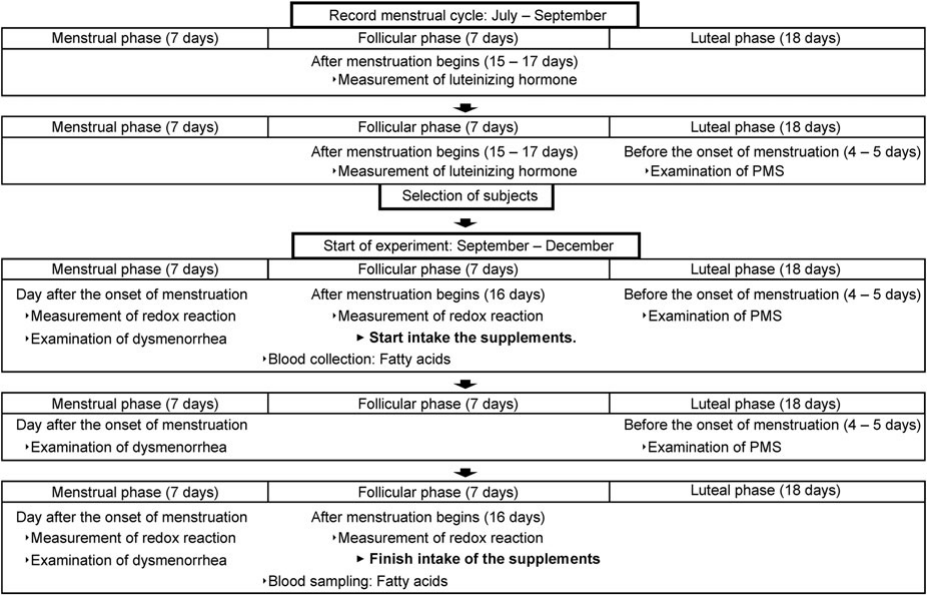
Experimental protocol. Case of the 32-day menstrual cycle.

The study was conducted as a randomized, double-blind, placebo-controlled trial. The trial period started from July to December. During the period from July to September (two menstrual cycles), the participants with a regular period were selected. The interventional trial was conducted between September and December, which corresponded to the two menstrual cycles. The experimental protocol is shown in Figure 2.

The participants were allocated into two groups, and stratified randomization was used to minimize bias in the amount of exercise due to the basketball club training. The EG and CG took the capsules for two menstrual cycles (about 8 weeks). The sequence allocation concealment and blinding of the participants and researchers were maintained throughout this period. To manage and record the supplement intake, participants noted the time of intake and health conditions on the record sheet.

### Redox reaction

Difference in reactive oxygen metabolites (d-ROMs), which indicate the degree of oxidative stress, and the biological antioxidant potential (BAP), which indicates antioxidant power, were measured by centrifuging and separating 50 μL of blood and using a free-radical analytical device (Free CarrioDuo, Wismerll Co., Tokyo, Japan). d-ROMs capture the hydroxy peroxide level in the plasma generated by active oxygen and free radicals in the body. d-ROMs were obtained by measuring hydroxy peroxide concentrations in a color reaction and calculating the change in absorbance. BAP was obtained by adding serum to a mixed solution of a reagent containing a thiocyanate derivative and a reagent containing iron ions and by calculating the change in absorbance when the serum was reduced from trivalent iron (Fe3+) to divalent iron (Fe2+).

The potential antioxidant capacity (BAP/d-ROMs) was calculated by dividing the BAP (indicates antioxidant power) by d-ROMs (indicates the degree of oxidation). The redox reaction was measured four times: during menstrual and follicular phases in two menstrual cycles.

### Blood collection

Under physician guidance, a nurse collected blood samples from the midline of the vein. Blood samples were collected after 8 hours of fasting. Four fractions of fatty acids from the participants were measured before and after the intervention. Approximately 8 mL of blood was drawn from the antecubital vein through a standard venipuncture technique. Blood was collected in ethylenediaminetetraacetic acid tubes for whole-blood and plasma analysis. Four fractions of fatty acids (arachidonic acid [AA], EPA, DHA, and EPA/DHA ratio) were measured to confirm EPA concentrations in the control and intervention groups; high EPA and DHA concentrations in the intervention group confirmed that the participants took the EPA/DHA supplemental capsule. In this study, 24 fractions of fatty acids in the serum were measured by LSI Medience Corporation (Sendai City, Japan).

### Questionnaire on PMS, dysmenorrhea, and nutritional intake

To assess the PMS, responses were “none, 0”; “mild, 1”; “medium, 2”; and “severe, 3” based on the questionnaire used in a previous study by Takeda et al.^19^ The total response score was 69 points. In addition, participants responded about dysmenorrhea symptoms (lower abdominal pain, lower back pain, etc.) using a numerical rating scale.

To confirm the nutritional intake of the participants, we used the Food Frequency Questionnaire Based on Food Groups version 5 (FFQg) software to conduct the food survey before and after the intake of the trial food.

### Statistical analysis

All data are expressed as the mean ± standard deviation. Significance between EG and CG was calculated using a two-way analysis of variance (ANOVA), and a two-way ANOVA with repeated measures in the groups (EG vs. CG) was used to test for the interaction and main effects. If a significant difference was noted in the interaction (condition × time), the Bonferroni method was used to perform multiple comparisons of all groups in subsequent tests. If a significant difference was only found in the main effect (time), the *t*-test was used. Furthermore, to show the effect size, a two-way ANOVA was used to calculate the partial eta squared (*η*^2^), and the *t*-test was calculated using Cohen's *d*. Unpaired *t*-test and redox reaction were used to compare the menstrual and follicular phases before the intake of the trial food. Statistics were calculated using the SPSS software; *p* < 0.05 was considered significant.

## Results

No significant difference was found in the characteristics between the two groups (EG, *n* = 11, age: 20.1 ± 1.1 years, height: 166.6 ± 3.8 cm, weight: 57.7 ± 4.5 kg, and body fat: 17.3% ± 2.7%; CG, *n* = 10, age: 19.9 ± 1.4 years, height: 165.6 ± 6.0 cm, weight, 59.2 ± 6.0 kg, and body fat: 18.7% ± 4.3%). Two subjects were excluded because they did not have regular menstrual cycles. Using the FFQg, no significant difference was noted in the nutrient intake between the two groups (EG, energy: 1506.3 ± 348.3 kcal, protein: 45.6 ± 12.7 g, fat: 47.6 ± 13.6 g, carbohydrate: 203.3 ± 49.3 g, and omega-3 fatty acid: 1.0 ± 0.4 g; CG, energy: 1349.4 ± 215.9 kcal, protein: 42.7 ± 11.6 g, fat: 42.5 ± 8.8 g, carbohydrate: 192.6 ± 34.4 g, and ome2ga-3 fatty acid: 1.0 ± 0.3 g). The nutritional intake of both the groups did not differ before and after the intervention.

### Redox reaction

The result of the two-way ANOVA on pre- and posttrial food d-ROMs, BAP, and BAP/d-ROMs for each cycle showed a significant difference in d-ROMs and BAP/d-ROMs during the menstrual phase by the main effect of time (before and after intake) for EG and CG (d-ROMs: *p* = 0.003, *η*^2^ = 0.370, BAP/d-ROMs: *p* = 0.022, *η*^2^ = 0.248). Subsequent tests showed a significant decrease in d-ROMS of EG and CG during the menstrual phase after an intake of the trial food (EG: *p* = 0.045, *d* = 0.689, CG: *p* = 0.036, *d* = 0.781); however, no significant difference was found in the BAP/d-ROMs after an intake of the trial food. In the follicular phase, significant differences were observed only in the main effects of d-ROMs and BAP/d-ROMs (d-ROMs: *p* = 0.008, *η*^2^ = 0.312, BAP/d-ROMs: *p* = 0.04, *η*^2^ = 0.204).

In the *post hoc* test, d-ROM and BAP/d-ROM of the EG were significantly different before and after supplementation (d-ROMs: *p* = 0.008, *d* = 1.008, BAP/d-ROMs: *p* = 0.026, *d* = −0.778). The above results are shown in Table 1.

**Table 1.** Effects of Supplementation and Redox Reactions During Each Menstrual Cycle

	EG pre	EG post	*p*	d	CG pre	CG post	*p*	d
Menstrual phase								
d-ROMs (CARR U)	291.2 ± 42.0	269.0 ± 37.6	0.045^*^	0.689	274.2 ± 33.6	251.7 ± 31.5	0.036^*^	0.781
	Time × condition interaction: *p* = 0.981 and *η*^2^ = 0.0; time main effect: *p* = 0.003 and *η*^2^ = 0.370							
BAP (μM)	2010.1 ± 142.2	2018.7 ± 155.0	—	—	2012.5 ± 117.8	1969.9 ± 85.0	—	
	Time × condition interaction: *p* = 0.300 and *η*^2^ = 0.121; time main effect: *p* = 0.489 and *η*^2^ = 0.0							
Antioxidant capacity (BAP/d-ROMs)	7.0 ± 1.0	7.6 ± 1.0	0.067	−0.619	7.5 ± 1.2	7.9 ± 0.9	0.166	−0.477
	Time × condition interaction: *p* = 0.778 and *η*^2^ = 0.004; time main effect: *p* = 0.022 and *η*^2^ = 0.248							
Follicular phase								
d-ROMs (CARR U)	301.0 ± 47.6	262.6 ± 39.7	0.008^*^	1.008	273.7 ± 24.8	257.6 ± 36.9	0.304	0.345
	Time × condition interaction: *p* = 0.245 and *η*^2^ = 0.071; time main effect: *p* = 0.008 and *η*^2^ = 0.312							
BAP (μM)	2015.6 ± 141.3	1970.8 ± 176.0	—	—	2103.9 ± 119.2	2073.3 ± 151.2	—	
	Time × condition interaction: *p* = 0.585 and *η*^2^ = 0.002; time main effect: *p* = 0.346 and *η*^2^ = 0.047							
Antioxidant capacity (BAP/d-ROMs)	6.8 ± 1.4	7.6 ± 1.3	0.026^*^	−0.778	7.7 ± 0.8	8.2 ± 1.5	0.368	−0.300
	Time × condition interaction: *p* = 0.590 and *η*^2^ = 0.016; time main effect: *p* = 0.040 and *η*^2^ = 0.204							

Data are presented as mean ± SD. Statistics: two-way ANOVA (*p* < 0.05). *Post hoc* test: paired *t*-test (*p* < 0.05). *d* and *η*^2^ value = effect size. Pre (before supplementation); post (after supplementation).

^*^ *p* < 0.05 a significant difference between groups.

ANOVA, analysis of variance; BAP, biological antioxidant potential; CARR U, Carratelli unit; CG, control group; d-ROMs, difference in reactive oxygen metabolites; EG, EPA/DHA group.

The results of the redox reactions between menstrual cycles were as follows: d-ROMs were 283 ± 38.3 Carratelli unit (CARR U) and 288 ± 40.0 CARR U during the menstrual and follicular phases, respectively. BAP levels were 1996 ± 137.8 and 2058 ± 135.7 μM during the menstrual and follicular phases, respectively, and the BAP/d-ROMs were 7.2 ± 1.1 and 7.2 ± 1.2 during the menstrual and follicular phases, respectively, and no significant difference was noted.

### PMS and dysmenorrhea

The results of PMS and dysmenorrhea were scored, and data were used in the two-way ANOVA. No significant difference was observed in the main effect or interaction (Table 2).

**Table 2.** Effects of Supplementation on Premenstrual Syndrome and Dysmenorrhea

	EG			CG		
	Pre	Post: Phase 1	Post: Phase 2	Pre	Post: Phase 1	Post: Phase 2
Premenstrual syndrome (point)	7.9 ± 6.1	7.6 ± 6.0	7.0 ± 5.9	10.4 ± 9.6	5.7 ± 5.2	7.4 ± 4.6
Time × condition interaction: *p* = 0.253 and *η*^2^ = 0.081; time main effect: *p* = 0.164 and *η*^2^ = 0.113						
Dysmenorrhea (point)	2.2 ± 1.3	1.9 ± 1.0	2.2 ± 1.7	2.5 ± 2.2	2.6 ± 2.8	2.2 ± 1.9
Time × condition interaction: *p* = 0.704 and *η*^2^ = 0.013; time main effect: *p* = 0.917 and *η*^2^ = 0.005						

Pre (before supplementation), post: phases 1 and 2 (menstrual weeks 1 and 2 after supplementation). Statistics: two-way ANOVA (*p* < 0.05).

### Four fractions of fatty acids

EPA (pre: 16.9 ± 6.7 μg/mL; post: 59.6 ± 34.8 μg/mL; *p* = 0.002), DHA (pre: 55.8 ± 18.7 μg/mL; post: 84.7 ± 20.6 μg/mL; *p* = 0.000), and EPA/AA ratio (pre: 0.09 ± 0.03; post: 0.31 ± 0.19; *p* = 0.003) of EG showed a significant difference after the intake of the trial food. However, no significant difference was noted in AA (pre: 181.6 ± 28.6 μg/mL; post: 204 ± 44.5 μg/mL; *p* = 0.064). AA (pre: 186.9 ± 47.6 μg/mL; post: 186.5 ± 27.7 μg/mL; *p* = 0.972), EPA (pre: 22.0 ± 9.9 μg/mL; post: 27.5 ± 20.6 μg/mL; *p* = 0.368), DHA (pre: 67.3 ± 19.5 μg/mL; post: 67.7 ± 22.8 μg/mL; *p* = 0.944), and EPA/AA ratio (pre: 0.12 ± 0.05; post: 0.15 ± 0.12; *p* = 0.366) of CG did not show a significant difference.

## Discussion

In this study, we compared d-ROMs, BAP, and BAP/d-ROMs before and after intake of the trial food for each menstrual cycle. d-ROMs during menstrual phase were significantly lower after an intake of the trial food for EG and CG. During the follicular phase, this trend was only seen in EG. BAP/d-ROMs during the follicular phase was significantly higher after an intake only in EG. In addition, BAP/d-ROMs during the menstrual phase showed a decreasing trend after an intake in EG. These results indicate that an EPA intake suppressed oxidation in the oxidation–reduction balance, leaning toward the reduction side. In addition, oxidative stress was reduced despite inadequate nutritional intake in participants.

A possible factor is the anti-inflammatory and physiological functions of EPA. EPA and DHA can partially inhibit inflammation: the production of eicosanoids, such as prostaglandins and leukotrienes, from n-6 fatty acid AA, and production of inflammatory cytokines.^20–22^ This means that these immune cells suppress excessive production of ROS, where muscle damage from muscle movement activates macrophages through the production of cytokines.^9,23^ Furthermore, anti-inflammatory actions following an exercise load were clarified by Corder, who conducted an intervention trial with women using omega-3 fatty acid DHA.^24^ Considering that this study's participants were athletes exposed to oxidative stress, a decrease in d-ROMs with an intake of EPA and DHA, the effects on BAP/d-ROMs can be explained with the above reasons. In the study by Buonocore et al., n-3 fatty acid supplementation was given to long-distance runners for 8 weeks.

As a result, oxidative and inflammatory markers decreased.^25^ The results of this study also showed a decrease in oxidative markers. Furthermore, in the inflammatory process during menstruation, a decrease in progesterone suppresses the metabolism of prostaglandins, eliminating the protective functions of ROS. When active oxygen increases, nuclear factor-κB changes from a restraint to a release state, activating the transcription of target genes and increasing the synthesis of inflammatory prostaglandins.^26,27^ As such, in an endocrine event of a menstrual cycle, the anti-inflammatory action of EPA is likely involved with the above-described mechanism. Furthermore, given that the lack of change in BAP indicates antioxidant effects, with the actions of EPA and DHA, a reducing agent was not needed.

However, in a previous study, its action as an antioxidant has been indicated.^11^ The above mechanism on EPA supplementation is the reason why the redox balance tilts toward reduction. A decrease in d-ROMs was confirmed in CG during the menstrual phase. Fluctuations in d-ROMs in both groups were lower after the intake of the trial food. This possibly occurred because of a hot environment and oxidative stress. For the measurement of redox reaction in the menstrual phase, preintake was conducted from early to late September, and postintake was given from late October to mid-December. During the former period, caution was taken in regard to heat stress. Heat stress is an environmental factor that could stimulate ROS generation,^28^ and during the study period, a hot environment may have some effects on the fluctuations of oxidative stress. Considering the objective of this study, this is one of the limitations.

In a previous study, the degree of oxidative stress gradually increased from the menstrual phase to the follicular phase^4,5^; however, in this study, a similar trend was not observed. The study participants were college athletes who are members of a university basketball team; thus, the generation of endogenous antioxidants by exercise was more activated in these athletes than in the general female population. In addition, lifestyle is related to factors of oxidative stress.^29^ Specifically, this study's population had a lower average nutritional intake than the requirement^30,31^; thus, we can assume that different lifestyles and nutritional intake affect oxidation–reduction balance. A challenge with the health management of female athletes is the “low energy availability,”^32^ where many participants fit this description.

However, during the experimental period, the participants maintained regular menstrual cycles and conditions. However, we assume that it has a minor effect on the hormone level, and estrogen and progesterone levels in menstrual cycle affect redox reactions. However, in this study, these levels were not measured. Thus, we were unable to further discuss hormone levels, which is another limitation.

Moreover, no significant difference was found between the groups in terms of dysmenorrhea and PMS scales. The participants were not selected based on the experience of dysmenorrhea and PMS; thus, our results were inconsistent with previous studies on dysmenorrhea. However, because prostaglandin secretion is involved in dysmenorrhea,^33^ given the physiological actions of EPA, it might lessen dysmenorrhea. Overall, this study is limited by the relatively small sample size. Thus, a further survey with larger sample size is necessary. Furthermore, the effect size was calculated due to the small sample size; however, the significance was weak. Regarding the markers of redox reactions, Fumiaki et al. believe that individual differences in blood iron levels may affect the accuracy of the d-ROMs test.^34^

However, in a study by Tomonori et al, the relationship of nontransferrin-bound iron (NTBI) and oxidative stress with cardiac burden in the general population was observed; their results revealed an independent relationship between NTBI and d-ROM.^35^ In terms of their subjects, these two studies have characteristics similar to the present study.^7,36^ Moreover, these two studies, most recent previous studies,^37^^,38^ and most other studies have not adjusted for confounding iron concentrations. Therefore, we did not measure iron concentrations. As per the hypothesis by Fumiaki et al, the unmeasured iron concentration may be a limitation. In the future, we hope that their hypothesis will be clarified.

The strength of this study was on the management of menstrual cycle of each participant, which was performed by joint researchers in cooperation with the participants. Thus, measurements of biochemical markers, questionnaire survey on dysmenorrhea, and other processes were performed appropriately. Furthermore, this is the first study to provide EPA and DHA to female athletes over 8 weeks (two menstrual cycles) and observed the redox reaction during each menstrual cycle. The present result could lead to a recommendation of EPA-rich fish oil in female athletes for conditioning management.

## Conclusions

Supplementation with EPA over two menstrual cycles is strongly involved in the antioxidant action during the menstrual and follicular phases. This is an important finding in terms of controlling the redox balance during menstrual cycles.

## Data Sharing Statement

The datasets generated during and/or analyzed during the current study are available from the corresponding author on reasonable request.

## Abbreviations Used

AA: arachidonic acid
ANOVA: analysis of variance
BAP: biological antioxidant potential
CARR U: Carratelli unit
CG: control group
DHA: docosahexaenoic acid
d-ROMs: difference in reactive oxygen metabolites
EG: EPA/DHA group
EPA: eicosapentaenoic acid
FFQg: Food Frequency Questionnaire Based on Food Groups version 5
NTBI: nontransferrin-bound iron
PMS: premenstrual syndrome
ROS: reactive oxygen species
